# Comparative Transcriptome Analysis Provides Insights Into the Mechanism by Which 2,4-Dichlorophenoxyacetic Acid Improves Thermotolerance in *Lentinula edodes*

**DOI:** 10.3389/fmicb.2022.910255

**Published:** 2022-06-20

**Authors:** Ruiping Xu, Shasha Zhou, Jiaxin Song, Haiying Zhong, Tianwen Zhu, Yuhua Gong, Yan Zhou, Yinbing Bian

**Affiliations:** Institute of Applied Mycology, College of Plant Science and Technology, Huazhong Agricultural University, Wuhan, China

**Keywords:** comparative transcriptome, *Lentinula edodes*, 2,4-dichlorophenoxyacetic acid, heat stress, fatty acid metabolism

## Abstract

As the widest cultivated edible mushroom worldwide, *Lentinula edodes* suffers serious yield and quality losses from heat stress during growth and development, and in our previous study, exogenous 2,4-Dichlorophenoxyacetic acid (2,4-D) was found to improve the thermotolerance of *L. edodes* strain YS3357, but the molecular mechanism remains unclear. Here, we explored the potential protective mechanism of exogenous 2,4-D against heat stress by transcriptome analysis. 2,4-D possible improve the thermotolerance of *L. edodes* through regulating antioxidant genes, transcription factors, energy-provision system, membrane fluidity, and cell wall remodeling. Furthermore, 2,4-D was also found to regulate the saturation levels of fatty acids and ATP content in *L. edodes* mycelium under heat stress. This study proposed a regulatory network of 2,4-D in regulating *L. edodes* response to heat stress, providing a theoretical basis for improving *L. edodes* thermotolerance, and facilitating the understanding of the molecular mechanism of exogenous hormones in alleviating abiotic stress damage to macrofungi.

## Introduction

As the most widely cultivated edible mushroom with the highest output in the world (Royse et al., 2017), *Lentinula edodes* provide valuable benefits for the biotransformation of agricultural residues, but its production can be significantly affected by thermal stress. Previous studies have reported that the fruiting bodies of *Agaricus bisporus* were obviously reduced and became smaller after exposure to high temperatures for a few days. Heat stress not only affects the growth rate of fungal mycelium but also causes changes in the cell wall integrity and the increase of membrane fluidity (Liu et al., 2017; Qiu et al., 2018). Additionally, high temperature causes changes in the extracellular metabolites of *Pleurotus ostreatus* to promote *Trichoderma asperellum* growth. Furthermore, high temperatures can cause the rotting of logs or bags of *L. edodes* infected with *Trichoderma* spp., leading to yield and quality losses (Cao et al., 2015; Wang et al., 2016). All these reports suggest that improving the thermotolerance of *L. edodes* will help the mushroom cultivation industry.

Previous studies have shown that Para-aminobenzoic acid (PABA) synthase, nitric oxide, and trehalose can reduce the accumulation of reactive oxygen species (ROS) to alleviate oxidative damage induced by heat stress, thereby improving the thermotolerance of a variety of macrofungi (Kong et al., 2012; Yin et al., 2015; Luo et al., 2021). In our previous studies, *LetrpE*, *LeYUCCA8*, *LeDnaJ07*, auxin, and 2,4-Dichlorophenoxyacetic acid (2,4-D) were found to participate in the response of *L. edodes* to heat stress (Zhou et al., 2018a; Wang et al., 2018b, 2020; Zhong et al., 2021). 2,4-D, a functional analogue of the auxin indole-3-acetic acid (IAA) is widely used as an IAA substitute because it can induce IAA like activities (Maher and Martindale, 1980). Meanwhile, 2,4-D is more stable than IAA under blue and ultraviolet light (Stasinopoulos and Hangarter, 1990), making it more compatible than IAA as an exogenous auxin. In previous studies, researchers have tried to improve the thermotolerance of crops by using auxins, such as IAA, 2,4-D, or the 1-naphthaleneacetic acid (NAA; Sakata et al., 2010; Ishida et al., 2016). In our previous work, exogenous 2,4-D was shown to improve the thermotolerance of *L. edodes* strain YS3357 by increasing the mycelial antioxidant enzyme activity and reducing the cellular lipid peroxidation degree (Zhou et al., 2018b), but the molecular mechanism remains to be elucidated.

The purpose of this study was to explore the molecular mechanism by which 2,4-D improves the thermotolerance of *L. edodes* by using 0.01 mM 2,4-D to pre-treat YS3357 strain, followed by analysis of the differentially expressed genes (DEGs) *via* transcriptome induced by 2,4-D under heat stress. The DEGs were found to be involved in regulating antioxidant genes, transcription factors, energy-provision system, membrane fluidity, and cell wall remodeling. Additionally, 2,4-D was observed to modify the saturation levels of fatty acids and ATP contents in *L. edodes* mycelium response to heat stress. This study not only explores he potential molecular mechanism of 2,4-D in regulating *L. edodes* response to heat stress but also identifies the saturation levels of fatty acids as the response of metabolites in this process.

## Materials and Methods

### Sample Preparation

The sawdust medium (78 g of hardwood sawdust, 20 g of wheat bran, 2 g of lime and 20 g of agar in 1 l of distilled water) was prepared as the basic medium. Six plates were added with a final concentration of 0.01 mmol/l 2,4-D (group of 24D_), and the other six were added with equal methanol (group of CK_). *L. edodes* wild strain YS3357 was cultivated for 9 days at 25°C in the two groups of medium. Then, three plates (means three repeats) of both two groups were treated at 40°C for 24 h (divided into groups of CK_HS and 24D_HS), and the remaining three plates of both groups continued to grow at 25°C for 24 h (divided into groups of CK_NH and 24D_NH). After cultivation, all the samples were frozen in liquid nitrogen and used for RNA extraction.

### cDNA Library Construction, RNA Sequencing, and Transcriptome Data Processing

Total RNA was extracted using the RNAiso Plus method (TAKARA, Shanghai, China), and the cDNA library was constructed and sequenced according to the manufacturer’s instructions (Illumina, San Diego, CA, United States). The 12 cDNA libraries were sequenced at Beijing Genomics Institute (BGI)-Shenzhen (Wuhan, China), with three independent biological replicates of CK_NH, CK_HS, 24D_NH, and 24D_HS to sequence the YS3357 samples. No less than 3GB of clean data was obtained for each sample, and all the RNA-seq raw data were uploaded to NCBI SRA with the BioProject number: PRJNA813710.

Data pre-treated and quality control, mapping the RNA-seq reads to the *L. edodes* genome, gene expression levels calculation, DEGs screening, and transcription factors (TFs) identifing were all conducted following the description of our previous study (Wang et al., 2018b). 12 raw datasets of all samples were pre-treated by Trimmomatic, it was used to filter the low-quality reads and duplicate sequences of the raw reads to obtain clean reads (Bolger et al., 2014). Clean reads were mapped to *L. edodes* reference genome using Hisat2 (Kim et al., 2015; Chen et al., 2016). The mapped clean reads count was evaluated by HTseq (Anders et al., 2015) and converted to reads per kilobase of exon per million mapped reads (RPKM) data through TBtools (Chen et al., 2020). EdgeR was used to screen differentially expressed genes (DEGs) with |log2 (fold change)| >1 and false discovery rate (FDR) < 0.01 (Chen et al., 2014). DEGs functional were annotated by Blast2GO (Conesa et al., 2005). Whether HS and 2,4-D significantly affected gene expression levels were analyzed by comparing the log2 ratio of CK_HS/CK_NH and 24D_ HS/24D_ NH samples, respectively. The functions of all the DEGs were further investigated by Gene Ontology (GO) and Kyoto Encyclopedia of Genes and Genomes (KEGG) pathway enrichment analysis (Kanehisa et al., 2004; Ye et al., 2006). Heatmaps were generated by TBtools (Chen et al., 2020).

The weighted gene co-expression network (WGCNA) analysis followed our previous method (Wang et al., 2019). In the present study, CK_NH, CK_HS, 24D_NH, and 24D_HS were considered as different sample traits. After screening the gene modules most associated with the target trait, the gene modules of the co-expression network were constructed using Cytoscape (Kohl et al., 2011). DEGs with the highest connectivity value were defined as hub nodes in the network.

### qRT-PCR Analysis

To validate the transcriptome sequencing results, nine genes were randomly selected for qRT-PCR analysis using AceQ qPCR SYBR Master SYBR (Vazyme, Nanjing, China), with the actin gene as the internal control. The results were analyzed using the CFX Connect Real-Time PCR system (BIO-RAD), and the relative expression was calculated using the 2^−ΔΔCT^ method (Livak and Schmittgen, 2002).

### Fatty Acid Extraction and Analysis

Fatty acid (FA) extraction followed a previously method (Welti et al., 2002). Fatty acid methyl esters (FAMEs) were analyzed by gas chromatography (GC, Beckamn Coulter) and identified by their retention times relative to the mix standard of 37 components of FAMEs. The chromatographic peak area was measured as the relative content of the FAMEs.

### Determination of ATP Content

The ATP content of each sample was measured using an ATP assay kit (Solarbio, Beijing, China) as instructed by the manufacturer. The ATP extract from the kit was used to lyse the mycelium, followed by centrifugation at 8000 g for 10 min at 4°C, transferring supernatant to another EP tube, adding 500 μl of chloroform and well shaking, centrifugation at 10000 g for 3 min at 4°C, collecting the supernatant to determine the ATP content for different treatment groups.

### Statistical Analysis

Data were expressed as mean and standard deviation (SD). The significant differences between analyzed samples were determined by one-way ANOVA and evaluated through Duncan’s multiple range test at *p* < 0.05.

## Results

### Transcriptome Analysis of the Response of DEGs to Heat Stress Under 2,4-D Treatment

**Figure 1:**
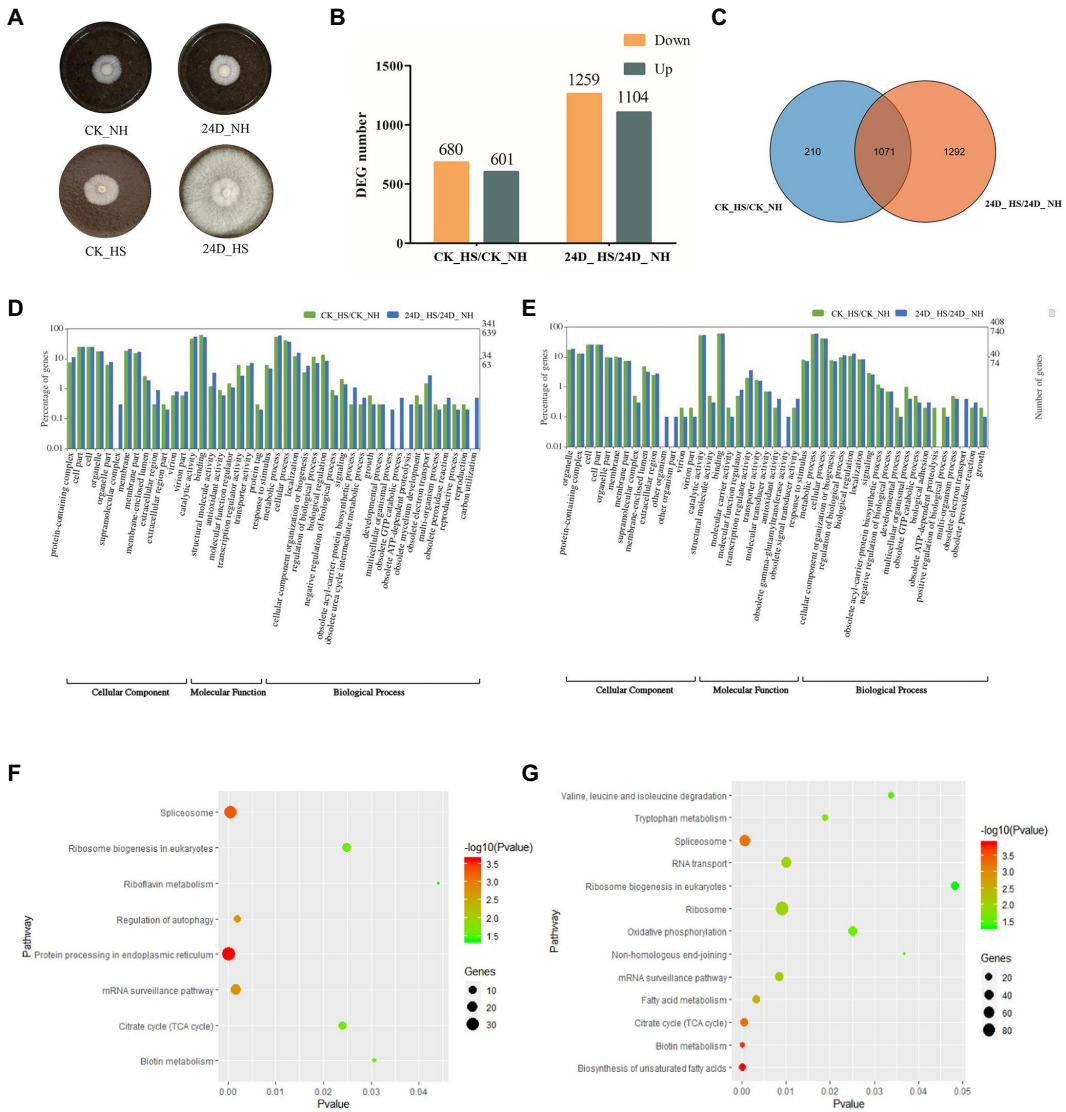
RNA-seq data analysis results of CK_HS/CK_NH and 24D_ HS/24D_ NH. **(A)** *L. edodes* mycelium cultured at 25°C for 6 days on sawdust medium, shown as CK_NH and 24D_ NH, followed by heat stress at 40°C for 24 h, and culture at 25°C for 15 days to resume growth, shown as CK_HS and 24D_ HS. **(B)** Column diagram for the number of DEGs. **(C)** Venn diagram for the number of DEGs. **(D)** GO term analysis results of up-regulated DEGs in CK_HS/CK_NH and 24D_ HS/24D_ NH. **(E)** GO term analysis results of down-regulated DEGs in CK_HS/CK_NH and 24D_ HS/24D_ NH. **(F)** KEGG pathway analysis results for CK_HS/CK_NH DEGs. **(G)** KEGG pathway analysis results for 24D_ HS/24D_ NH DEGs. CK_NH: no heat stress; CK_HS: heat stress; 24D_NH: exogenous 2,4-D without heat stress; 24D_HS: exogenous 2,4-D with heat stress. DEGs, differentially expressed genes.

In our previous study, 2,4-D was found to improve the thermotolerance of the *L. edodes* strain YS3357 (Figure 1A; Zhou et al., 2018a). To obtain an overview of the *L. edodes* transcriptomic responses to 2,4-D treatment under heat stress, CK_NH, CK_HS, 24D_NH, and 24D_HS samples with 12 RNA-Seq cDNA libraries, obtaining 20,566,345, 19,509,383, 20,336,913 and 20,310,599 150-bp paired-end reads for the four groups of samples, respectively, with Q30 > 94.93% for clean reads. Using the splice-aware aligner Tophat2, about 60% of the reads in YS3357 were mapped to the *L. edodes* (W1-26) genome sequence (Supplementary Table S1), and the matching rate was related to the genetic characteristics of different strains: W1 (W1-26 parent) is a cultured strain, while YS3357 is a wild-type strain. The Pearson correlation coefficients of different replicates per group were calculated and their values were over 86% (YS3357) in each sample (Supplementary Figure S1), indicating that the sequencing results were reliable and could be used for subsequent analysis.

For better understanding of the biological mechanism of 2, 4-D in enhancing the thermotolerance of *L. edodes* mycelium, we analyzed the DEGs in CK_HS/CK_NH and 24D_ HS/24D_ NH, with 1,281 and 2,363 DEGs (more than 2-fold or less than 0.5-fold) identified in the two groups, respectively. The number of DEGs between the two groups were shown in Figures 1B,C. These DEGs were considered as important candidate genes response to the heat stress by 2,4-D and used for further analysis.

The up-regulated and down-regulated DEGs in CK_HS/CK_NH and 24D_ HS/24D_ NH were classified by GO term annotation. The up-regulated DEGs in the two groups showed the top three enriched terms, the categories of cellular component (catalytic activity, cell part, and organelle), molecular function (binding, transcription regulator activity, and transporter activity), and biological process (metabolic process, cellular process, and localization; Figure 1D). Among the down-regulated DEGs in the two groups, the top three enriched terms included cellular component (catalytic activity, cell part, and cell), biological process (metabolic process, response to stimulus, cellular process, and biological regulation), and molecular function (binding, transcription regulator activity, and transporter activity; Figure 1E).

Based on the GO annotation of DEGs, the up-regulated genes of 24D_ HS/24D_ NH showed unique functions in supramolecular complex, multicellular organismal process, obsolete GTP catabolic process, obsolete ATP-dependent proteolysis, and carbon utilization (Supplementary Table S2), while downregulated genes showed unique functions in the other organism, other organism part, obsolete gamma-glutamyltransferase activity, and obsolete electron transport (Supplementary Table S3).

The functions of 2,4-D in enhancing the thermotolerance of *L. edodes* were further investigated by KEGG pathway analysis induced by 2,4-D and heat stress exposure. Between CK_HS/CK_NH, 762 DEGs were enriched in 107 KEGG pathways, and between 24D_ HS/24D_ NH, 1876 DEGs were enriched in 115 KEGG pathways. KEGG pathway enrichment analysis revealed that 24D_ HS/24D_ NH was different from CK_HS/CK_NH in most of the enrichment pathways, such as tryptophan metabolism, oxidative phosphorylation, biosynthesis of unsaturated fatty acids, FA metabolism, non-homologous end-joining, ribosome, RNA transport, and the degradation of valine, leucine and isoleucine (Figures 1F,G). These different enrichment pathways were considered as essential for improving mycelial thermotolerance.

The RNA-seq data were confirmed by qRT-PCR analysis of the expression of nine randomly selected genes (Supplementary Table S4). The information for each gene and primer is shown in Supplementary Table S4. Correlation analysis was performed on gene folding changes between the two treatment groups (RNA-seq and qRT-PCR). The qRT-PCR data were seen to agree with the RNA-seq data, with a significant positive correlation (*R*^2^ = 0.8918), indicating the reliability of the RNA-seq data (Supplementary Figure S2A). We selected four genes with significant difference expression at 24D_ HS/24D_ NH for the figure, which were relatively consistent with the transcriptome data (Supplementary Figure S2B).

### 2,4-D Regulated the Expression of Antioxidant Genes in Response to Heat Stress

Oxidative stress was reported directly induce cell damage after heat stress (Luo et al., 2021). Here, transcriptome analysis showed that the expressions of genes encoding ROS scavenging enzymes were significantly changed by exogenous addition of 2,4-D under heat stress, and we identified the genes encoding such enzymes, including catalase (CAT), glutathione-S transferase (GST), ascorbate peroxidase (APX), lipoxygenase (LOX), and thioredoxin (TRX).

**Figure 2:**
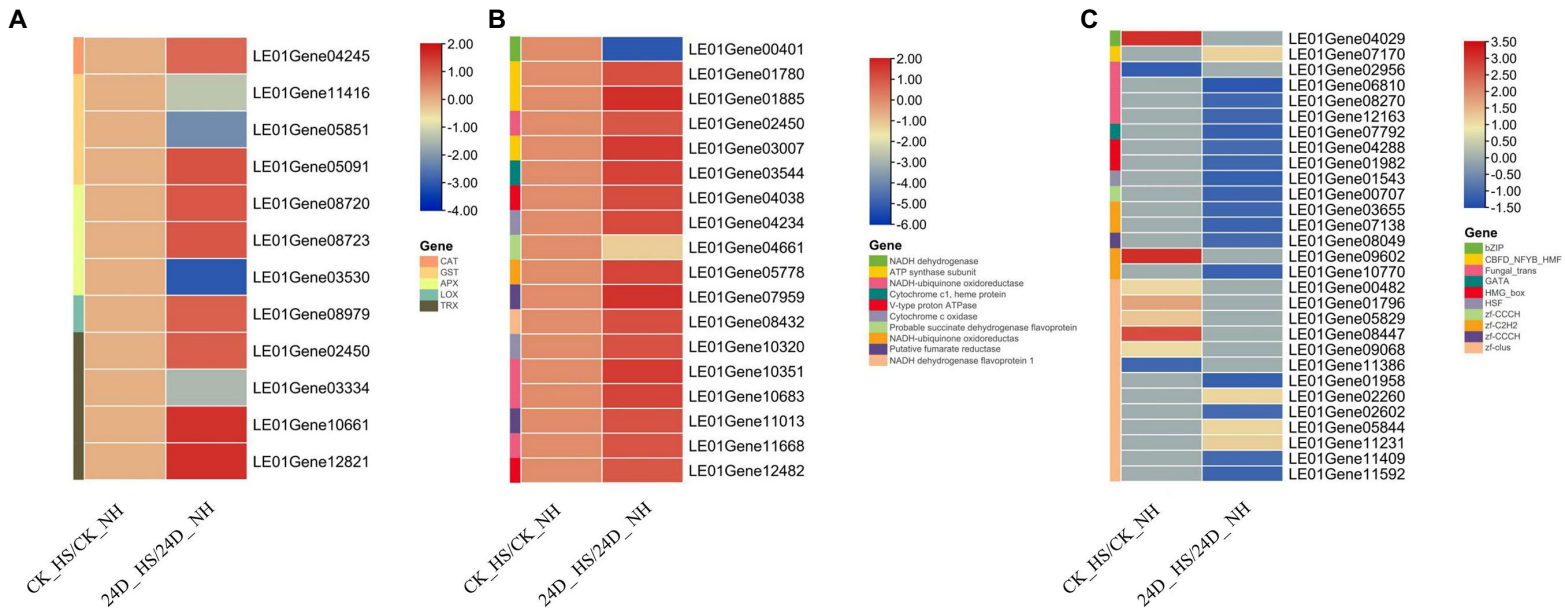
Heat map diagram showing CK_HS/CK_NH and 24D_ HS/24D_ NH related differentially expressed genes (DEGs). **(A)** Effects of 2,4-D and heat stress on the expression of antioxidant genes. **(B)** Effects of 2,4-D and heat stress on the expression of oxidative phosphorylation related genes. **(C)** Effects of 2,4-D and heat stress on the expression of transcription factors. Genes with no differential expression are indicated by 0 in the heatmap.

Among them, one APX gene (LE01Gene03530), two GST genes (LE01Gene11416 and LE01Gene05851), and one TRX gene (LE01Gene03334) were not differentially expressed between CK_HS/CK_NH but down-regulated by 2,4-D under heat stress. Additionally, the following genes were not differentially expressed between CK_HS/CK_NH but up-regulated between 24D_ HS/24D_ NH, such as two APX genes (LE01Gene08720 and LE01Gene08723), one GST gene (LE01Gene05091), one CAT gene (LE01Gene04245), one LOX gene (LE01Gene08979), and five TRX genes (LE01Gene02450, LE01Gene04578, LE01Gene10661, LE01Gene12376, LE01Gene12821; Figure 2A). These results suggested that 2,4-D may improve *L. edodes* thermotolerance by regulating the expression of antioxidant genes.

### 2,4-D Regulated Oxidative Phosphorylation in Response to Heat Stress

As previously reported, mitochondria are important organelles for ATP production in eukaryotic cells and oxidative phosphorylation-dependent energy conversion, including the respiratory chain and ATP synthase (Suhm and Ott, 2016). Moreover, oxidative phosphorylation plays a very important role in cellular stress response (Jin et al., 2021). KEGG analysis revealed the enrichment of oxidative phosphorylation in the 24D_ HS/24D_ NH group, and analyzing the expression of genes related to oxidative phosphorylation. A total of 18 oxidative phosphorylation-related genes were found to be differentially expressed under 2,4-D exposure and heat stress (Figure 2B), with only two genes (LE01Gene00401 and LE01Gene04661) were down-regulated in 24D_ HS/24D_ NH but not differentially expressed in CK_HS/CK_NH, 16 genes up-regulated in 24D_ HS/24D_ NH but not differentially expressed in CK_HS/CK_NH. Furthermore, we measured the ATP content of CK_NH, CK_HS, 24D_NH, and 24D_HS, and the ATP content was found to increase significantly under heat stress, but this increase was significantly inhibited by exogenous 2,4-D (Supplementary Figure S3). These results indicated that 2,4-D may improve *L. edodes* thermotolerance by regulating oxidative phosphorylation-related genes and ATP production.

### 2,4-D Regulated the Expression of Transcription Factors in Response to Heat Stress

Transcription factors (TFs), the essential regulators of gene expression in a cell, play important roles in regulating responses to diverse abiotic stresses and activating the downstream targets to improve stress tolerance (John et al., 2021), and they are also emerging as key regulatory elements of fungal gene expression (Shelest, 2008). Interestingly, 30 TFs were differentially expressed between CK_HS/CK_NH and 24D_ HS/24D_ NH, including bZIP, zf-C2H2, zf-clus, Fungal_trans, HMG_box, and other TF family members (Figure 2C). These TFs were not induced by heat stress, but by 2,4-D under heat stress, may participate in *L. edodes* thermotolerance.

### 2,4-D Regulated IAA Biosynthesis Pathway in Response to Heat Stress

In the present study, the effects of 2,4-D on the endogenous auxin biosynthesis pathway in improving *L. edodes* thermotolerance were evaluated by analyzing the expression of genes related to auxin biosynthesis. Combing the study in *Magnaporthe oryzae* and genomic analysis of *L. edodes* (Dong et al., 2022), we deduced three possible growth hormone synthesis pathways in *L. edodes*, including the YUC, IPyA and TPA pathways (Supplementary Figure S4A).

In the transcriptome data, we observed that three related genes were differentially expressed under 2,4-D and heat stress. Specifically, indole pyruvate decarboxylase genes *ipdc-1* (LE01Gene11499) and *ipdc-2* (LE01Gene12136), components of the IPyA pathway, were not differentially expressed between CK_HS/CK_NH but down-regulated between 24D_ HS/24D_ NH. *ALDH* (LE01Gene12649), encoding aldehyde dehydrogenase, was not differentially expressed between CK_HS/CK_NH but up-regulated between 24D_ HS/24D_ NH (Supplementary Figure S4B). These results indicated that 2,4-D may influence the thermotolerance of *L. edodes* by regulating the auxin biosynthesis pathway.

### 2,4-D Regulated Genes Involved in TCA Cycle and Glycolysis in Response to Heat Stress

**Figure 3:**
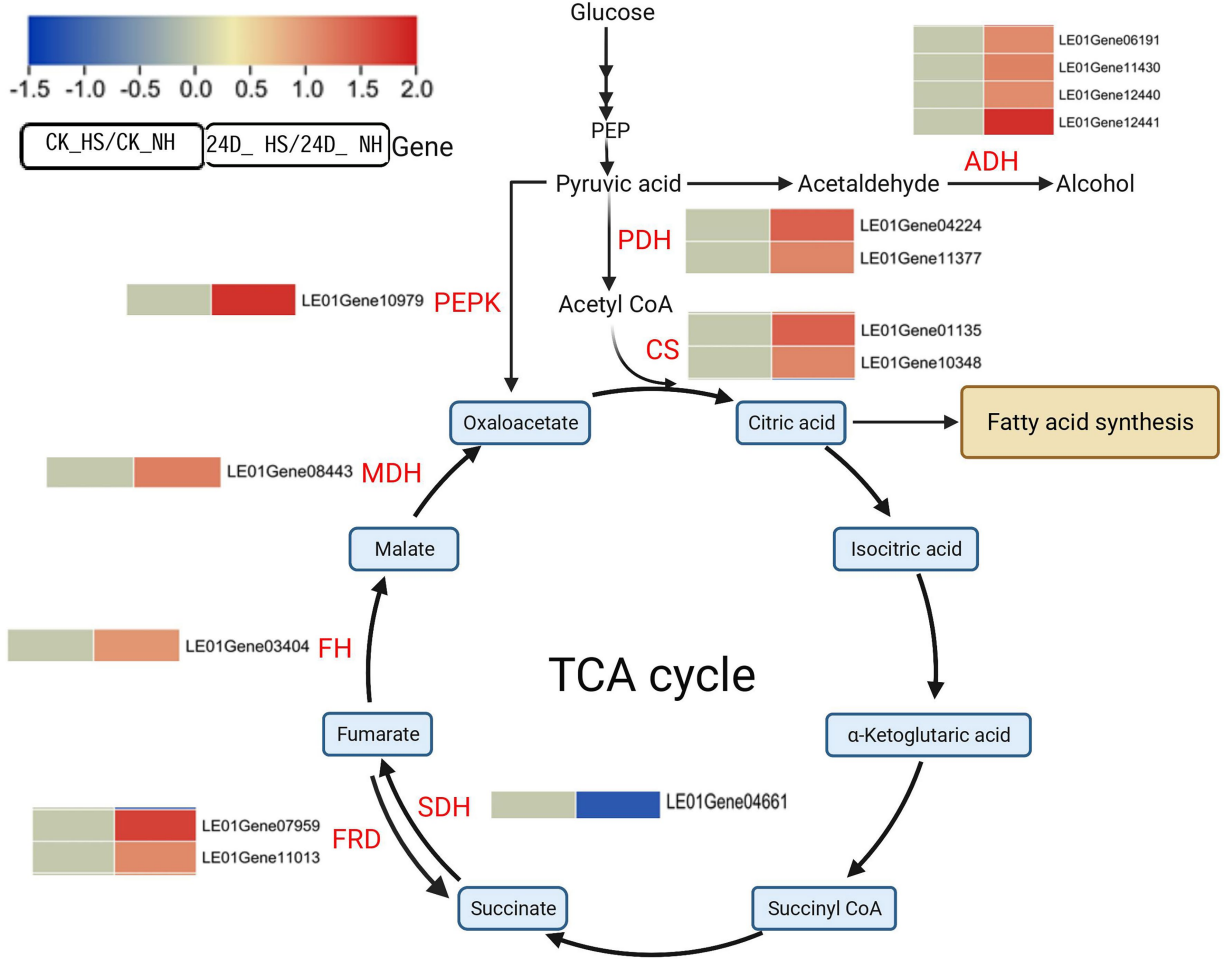
Effects of 2,4-D and heat stress on the expression of genes related to the TCA cycle. Enzyme names are in red font and metabolites are in black font. Red and blue squares represent up-regulation and down-regulation, respectively. Solid lines and multiple solid arrows indicate single- and multi-step reactions, respectively. Beside the name of the enzyme is the corresponding gene ID. The same row in the heat map indicates changes in gene abundance in CK_HS/CK_NH (left column) and 24D_ HS/24D_ NH (right column), followed by the gene ID. Genes with no differential expression are indicated by 0 in the heatmap. PEPK, phosphoenolpyruvate carboxykinase; ADH, alcohol dehydrogenase; PDH, pyruvate dehydrogenase; CS, citrate synthase; SDH, succinate dehydrogenase; FRD, fumarate reductase; FRD, fumarate reductase; FH, fumarate hydratase; MDH, malate dehydrogenase. This graph is created with biorender.com.

KEGG enrichment analysis showed that TCA cycle-related genes were related to 2,4-D response under heat stress, and further analysis revealed that 10 genes were differentially expressed in 24D_ HS/24D_ NH, but not differentially expressed in CK_HS/CK_NH, including nine up-regulated genes (LE01Gene01135, LE01Gene03404, LE01Gene04224, LE01Gene07959, LE01Gene08443, LE01Gene10348, LE01Gene10979, LE01Gene11013, LE01Gene11377), and one down-regulated gene (LE01Gene04661; Figure 3). Increasing the expression of these genes accelerates the TCA cycle and generates energy, notably phosphoenolpyruvate carboxykinase (PEPK), which produces oxaloacetate from pyruvate to sustain the TCA cycle.

Pyruvate is a key metabolite linking glycolysis and the TCA cycle (Fernie et al., 2004), and the enzyme genes in the glycolytic pathway from glucose to pyruvate were not differentially expressed in CK_HS/CK_NH and 24D_ HS/24D_ NH. Under aerobic respiration conditions, pyruvate can be converted to acetyl CoA through pyruvate dehydrogenase, while under anaerobic respiration, pyruvate can produce lactate or ethanol through lactate dehydrogenase and ethanol dehydrogenase, respectively (Sakihama et al., 2019). In the transcriptome results, we found four alcohol dehydrogenase (ADH) genes (LE01Gene06191, LE01Gene11430, LE01Gene12440, LE01Gene12441) up-regulated between 24D_ HS/24D_ NH but not differentially expressed between CK_HS/CK_NH (Figure 3), with the lactate dehydrogenase genes being not differentially expressed in either 24D_ HS/24D_ NH or CK_HS/CK_NH, leading to a possible reduction in the acetaldehyde contents. We also found the pyruvate dehydrogenase (PDH) genes were not differentially expressed between CK_HS/CK_NH but were up-regulated between 24D_ HS/24D_ NH (Figure 3).

Citrate synthase (CS) is the key rate-limiting enzyme in the TCA cycle (Li et al., 2018b), and we observed that citrate synthase genes (LE01Gene01135 and LE01Gene10348) were up-regulated in 24D_ HS/24D_ NH, but not differentially expressed in CK_HS/CK_NH (Figure 3). These results suggested that 2,4-D may improve *L. edodes*’ thermotolerance by regulating the key genes related to the TCA cycle.

### 2,4-D Regulated the Fatty Acid Saturation Level in Response to Heat Stress

In the KEGG pathway analysis, we found the enrichment of the biosynthesis of unsaturated FAs and FA metabolism in 24D_ HS/24D_ NH, and Supplementary Table S5 shows the changes in transcript levels of key genes in CK_HS/CK_NH and 24D_ HS/24D_ NH. Peroxisome plays an essential part in cellular metabolism, including FA oxidation (Van Roermund et al., 2003). In this study, we found that seven peroxisome-related genes were not differentially expressed between CK_HS/CK_NH, but were up-regulated between 24D_ HS/24D_ NH (Supplementary Table S6), suggesting that 2,4-D may regulate the FA metabolism under heat stress.

**Figure 4:**
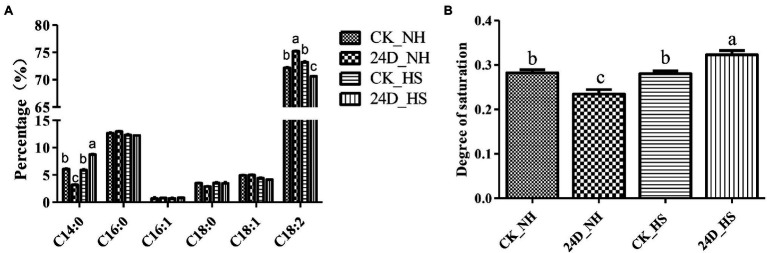
Analysis of fatty acid composition and saturation degree of CK_NH, CK_HS, 24D_NH, and 24D_HS samples. **(A)** Percentage for each fatty acid content obtained by normalization with internal standards. **(B)** The saturation degree of fatty acids determined by the rate of saturated to unsaturated fatty acids. Total unsaturated fatty acids to the sum of C16:1, C18:1, and C18:2; total saturated fatty acids to the sum of C14:0, C16:0, and C18:0. Different small letters indicate significant differences between samples (*p* < 0.05).

At the same time, in order to explore whether the FA content of *L. edodes* in response to 2,4-D under heat stress, we measured the FA content in CK_NH, 24D_NH, CK_HS, and 24D_HS by GC–MS. The results showed that the FA saturation degree changed significantly in 24D_HS versus CK_HS (Figure 4A). Interestingly, compared with CK_NH, 24D_NH showed a significant decrease in the FA saturation degree, suggesting that the addition of 2,4-D affected the saturation degree of FA, while CK_HS showed no significant change compared to CK_NH (Figure 4B). The results indicate that 2,4-D can regulate the FA saturation degree under heat stress, and has a potential relationship with *L. edodes* thermotolerance.

### Co-expression Network Analysis Obtain Key Genes in 24D_HS

The soft threshold was screened as 14, and the genes with different expression patterns were clustered into 11 modules and distinguished by different color blocks (Supplementary Figure S5A) The module with the highest correlation to the 24D_HS was MEbrown (Supplementary Figure S5B). A total of 877 genes were clustered in MEbrown module, with 141 genes in the co-expression network. The top three hub genes, co-expression networks, and GO terms are presented in Supplementary Figures S5C,D.

The top three hub genes (LE01Gene01937, LE01Gene05096, and LE01Gene05680) encode sulfate adenylyltransferase, condensin complex, and ubiquinol-cytochrome C reductase core protein 2, respectively. GO term analysis revealed that the 141 genes in the co-expression network were mainly enriched in cell part, organelle, protein-containing complex, and membrane part in the term of cellular component; binding and catalytic activity in the term of molecular function; metabolic process, cellular process, and localization in the term of biological process (Supplementary Figures S5C,D).

## Discussion

### 2,4-D Affects Antioxidant Genes in *Lentinula edodes* Heat Stress

Heat stress was reported to cause excessive ROS accumulation, DNA damage, protein denaturation, damage to lipid membrane permeability, and changes in cell wall integrity (Li et al., 2018a; Lippmann et al., 2019). High temperature could increase the auxin content in plants, so auxin plays a significant role in regulating plant adaptation to high temperature through auxin biosynthesis, signaling, and transport pathways (Franklin et al., 2011). As reported previously, the application of IAA, NAA, or 2,4-D could completely reverse male sterility in barley and *Arabidopsis* (Sakata et al., 2010). The pre-treatment of pea plants with auxins TA-12 and TA-14 could mitigate the oxidative stress provoked by high-temperature treatment (Sergiev et al., 2018). This is consistent with our previous results: (1) exogenous addition of IAA could significantly increase the thermotolerance of heat-sensitive strain YS3357, with a significant increase in the IAA content of mycelium under heat stress, and higher endogenous IAA content in the heat-tolerant strain than the heat-sensitive strain (Wang et al., 2018b, 2020); (2) exogenous 2,4-D could improve the thermotolerance of *L. edodes* strain YS3357 by increasing mycelial antioxidant enzyme activity and reducing cellular lipid peroxidation degree (Zhou et al., 2018b).

In this study, 2,4-D was found to regulate the expression of antioxidant genes to alleviate *L. edodes* heat stress. Based on RNA-seq data, exogenous application of 2,4-D can change the expression of *Trx* genes under heat stress. TRX is an important functional protein involved in scavenging intracellular ROS toxicity. Thioredoxin is a biomarker for oxidative stress, and many studies have shown the involvement of thioredoxins in a variety of redox-dependent cellular processes, such as gene expression, signal transduction, cell growth, and apoptosis (Matsuo and Yodoi, 2013). Trx2 was reported to be essential for maintaining the redox status when *Schizosaccharomyces pombe* was exposed to starvation and mild-heat stresses, indicating that cytoplasmic thioredoxins have an important role in adaptation to environmental change (Yano et al., 2010; Oku et al., 2013). These results indicated that 2,4-D may improve *L. edodes* thermotolerance through anti-oxidation reactions.

Collectively, exogenous 2,4-D may regulate the expression of *CAT*, *GST*, *APX*, *LOX*, and *TRX* genes to enhance the detoxification effect of ROS and reduce cell damage and cell death. As an antioxidant defense system, 2,4-D may improve the thermotolerance of *L. edodes* by scavenging active oxygen.

### 2,4-D Regulates Energy-Provision System and Fatty Acid Metabolism in *Lentinula edodes* Under Heat Stress

Stresses cause high energy consumption (De Block et al., 2005), leading to cell damage and energy imbalance under prolonged heat stress. The typical energy-provision pathways such as glycolysis, TCA cycle, and oxidative phosphorylation are cellular energy metabolic pathways, providing ATP for plant growth or defense (Fernie et al., 2004; Chen and Russo, 2012). As previously reported, heat stress could down-regulate energy metabolisms, decreasing the intermediate product of glycolysis and TCA cycle in the response of *L. edodes* to heat stress (Zhao et al., 2019). Exogenous L-Arginine was reported to enrich the DEGs involved in glycolysis, TCA cycle, and oxidative phosphorylation in *Gracilariopsis lemaneiformis* under heat stress (Zhang et al., 2021b). In the present study, 2,4-D could remarkably enhance the energy metabolism by activating the expression of genes encoding the TCA cycle (e.g., pyruvate dehydrogenase, citrate synthase, phosphoenolpyruvate carboxykinase, and fumarate reductase) and electron transport chain NADH dehydrogenase, cytochrome c reductase, cytochrome c oxidase, and ATPase (Figures 2B, 3) under thermal stress, it can provide energy for subsequent mycelial regeneration. Additionally, ATP content analysis showed that there may be a potential correlation between ATP content and 2,4-D to alleviate mycelial heat stress.

Pyruvate can be metabolized to lactate and ethanol in the glycolytic pathway, resulting in the accumulation of acetaldehyde as a DNA-damaging metabolite in the pathway to ethanol (Noguchi et al., 2016). In this study, we found that pyruvate decarboxylase and ethanol dehydrogenase were up-regulated while lactate dehydrogenase remained unchanged under 2,4-D exposure heat stress, which may have led to a reduction in acetaldehyde content, thus reducing cellular toxicity under heat stress. Meanwhile, pyruvate metabolism produces less energy during anaerobic respiration, but this energy is crucial for cell survival. The expression of these genes induced by 2,4-D at heat stress may play an important role in the recovery of *L. edodes* mycelium from thermal damage.

Citrate synthase, a central enzyme in carbon metabolism, is essential in the TCA cycle, amino acid synthesis, and the glyoxalate cycle. As a first step of the TCA cycle, citrate synthase catalyzes the condensation of oxaloacetate and acetyl-CoA to form citric acid (Ren et al., 2020). Citric acid could improve thermotolerance in *P. ostreatus* mycelium, nitric oxide (NO) inhibiting the expression of aconitase gene, resulting in citric acid accumulation under heat stress, inducing the expression of *Aox* gene to activate the alternative oxidative pathway (Hou et al., 2021). Based on transcriptome data analysis, under heat stress, 2,4-D could induce the up-regulated expression of two citrate synthase genes and *Aox* gene agreeing with NO alleviating heat stress damage in *P. ostreatus* mycelia, inferring that 2,4-D may regulate energy metabolism to improve the thermotolerance of *L. edodes.*

Moreover, citric acid is defined as a key precursor in lipid accumulation (Magdouli et al., 2020). In our study, three delta 12 desaturase genes showed up-regulated expression under 2,4-D exposure heat stress. Interestingly, we measured the FA content and found an increase in the FA saturation degree at 24D_HS, it probably due to variations in FA metabolism. As the key genes for FA β-oxidation, 2,4-D under heat stress induced the up-regulated expression of ketoacyl-CoA thiolase (LE01Gene02324), acylcarnitine carrier protein (LE01Gene12299), and hydroxyacyl-CoA dehydrogenase (LE01Gene07809; Supplementary Table S5). The saturation level of fatty acids may be an important factor in controlling plant thermotolerance (Larkindale and Huang, 2004; Zhang et al., 2021a), which was consistent with our results. Additionally, the expression of peroxisome related genes was up-regulated, promoting FA β-oxidation while producing a large amounts of acetyl-CoA, contributing to the TCA cycle. Our FA data suggested that the saturation levels of fatty acids were regulated by 2,4-D under heat stress, it could be a potential factor for the *L. edodes* mycelium to improve thermotolerance.

### 2,4-D Serves as a Regulator of Transcription Factors Involved in Heat Stress

Transcription factors play an important role in controlling various aspects of fungal metabolism, development, stress tolerance, members of the Zn2Cys6, C2H2, GATA, bZIP, and heat shock factor transcription factor (HSF) families were involved in abiotic stress response (John et al., 2021). HSFs with the HSF structural domain are regulators of the fungal heat shock protein (HSP) genes, to which the structural domain binds in response to heat shock and other stresses (Zhou et al., 2018a). In the present study, we found nine TF families between CK_HS/CK_NH and 24D_ HS/24D_ NH, with a total of 30 genes differentially expressed, including 21 TFs not induced by heat stress, but differentially expressed under 2,4-D exposure heat stress, such as Fungal_trans, GATA, zf-C2H2, and zf-clus and other TFs (Figure 4). Notably, the expression of DEGs involved in HSF was down-regulated in 24D_ HS/24D_ NH, but with no differential expression in CK_HS/CK_NH, indicating the negative regulation of HSF in *L. edodes* mycelium thermotolerance, inferring that the regulation of TFs on HSF may contribute to 2,4-D induced thermotolerance in *L. edodes*.

### 2,4-D May Improves *Lentinula edodes* Thermotolerance Through a Complex Network

Heat stress has emerged as one of the most destructive abiotic stresses on crops (Glazebrook, 1999). For macrofungi, heat stress may affect the production of fruiting bodies and even cause significant yield decrease (Lu et al., 2014). The effects of heat stress on fungi mainly include mycelial growth, cell wall structure, and membrane fluidity (Luo et al., 2021). Many previous studies have revealed the important roles of auxin and auxin signaling in improving the abiotic stress of plants (Piotrowska-Niczyporuk et al., 2018; Sergiev et al., 2018; Wang et al., 2021). However, the signal transduction pathway of auxin in fungi remains unknown.

**Figure 5:**
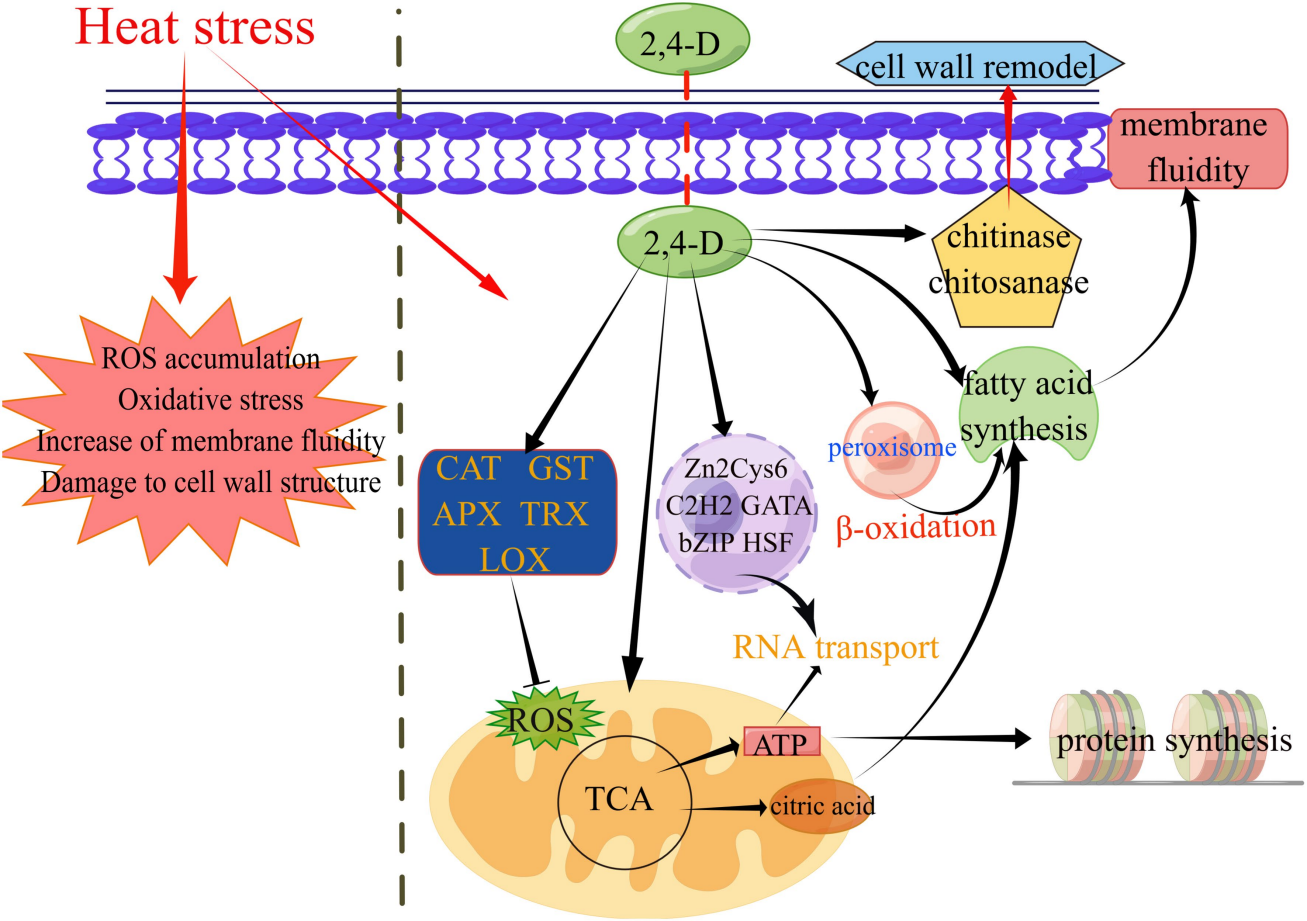
A proposed working model for the molecular mechanism of 2,4-D in enhancing *L. edodes* thermotolerance. This graph was generated by figdraw (www.figdraw.com).

In this study, the molecular role of 2,4-D in enhancing the thermotolerance of *L. edodes* mycelium was demonstrated by a complex regulatory network in combination with our findings (Figure 5). KEGG pathway analysis revealed Ribosome and RNA transport pathways exclusively in 24D_ HS/24D_ NH, suggesting that the 2,4-D may improve protein synthesis and increases the ability of RNA transport under heat stress. In eukaryotes, the spatio-temporal articulation of gene expression is mainly determined by the intertwined pathways of RNA transport and local translation regulation (Kindler et al., 2005).

For ROS elimination, 2,4-D can perform the functions of regulating antioxidant genes in heat stress (*CAT*, *GST*, *APX*, *LOX*, *TRX*). Heat stress can induce an increase in the level of endogenous IAA in *L. edodes* mycelium (Wang et al., 2018a). 2,4-D can also regulating the expression of auxin biosynthesis pathway-related genes under heat stress, thereby affecting downstream genes regulated by auxin. 2,4-D inducing the expression of some transcription factors (bZIP, zf-C2H2, zf-clus, etc.) to resist heat stress. 2,4-D inducing the expression of key genes involved in the TCA cycle, glycolysis and oxidative phosphorylation (citrate synthase, pyruvate dehydrogenase, alcohol dehydrogenase, etc.) under heat stress, thus affecting ATP production and FA synthesis.

Heat stress can also disrupt the integrity of the cell wall and damage the cell membrane, thereby altering the function of the plasma membrane structure and leading to increased membrane fluidity and reduced permeability (Ashraf and Harris, 2004). However, exogenous 2,4-D can reduce the membrane fluidity of cells through increase FA saturation degree in response to heat damage under heat stress. Micro-tubules are cytoskeletal elements and the αβ tubulin heterodimer is a structural subunit of micro-tubules essential for intracellular transport and cell division in all eukaryotes (Nogales et al., 1998). In GO annotation, the up-regulated genes in unique GO term enrichment at 24D_ HS/24D_ NH included *α-tubulin* and *β-tubulin* (Supplementary Table S2), suggesting that tubulin is involved in 2,4-D induced thermotolerance of *L. edodes*. Fungal chitinases are hydrolytic enzymes responsible for the degradation of chitin, with chitinases and chitosanase playing an essential role in cell wall remodeling and cell wall synthesis, respectively (Seidl, 2008). In the transcriptome data, the two enzyme genes were both up-regulated in 24D_ HS/24D_ NH, suggesting 2,4-D may be involved in cell wall remodeling after heat stress (Supplementary Table S7).

For the three hub genes in the co-expression network in 24D_HS, sulfate adenylyltransferase (LE01Gene01937) was the key energy gene of four moderate thermophiles and catalyze adenosine phosphosulfate (APS) to generate ATP and sulfate, which is the final stage of the sulfite oxidation so as to obtain energy (Zhou et al., 2015); the function of condensin complexes (LE01Gene05096) is versatile in gene regulation and chromosome segregation (Csankovszki et al., 2009); ubiquinol-cytochrome C reductase core protein 2 (LE01Gene05680) is a key subunit of the mitochondrial electron transport chain complex III (Sun et al., 2003). Collectively, these genes in MEbrown module may contribute to the 2,4-D induced thermotolerance of *L. edodes*.

## Conclusion

As an analogue of the endogenous auxin IAA, 2,4-D plays an important role in regulating plant responses to abiotic stresses. The content of endogenous IAA in *L. edodes* can affect its thermotolerance, and exogenous 2,4-D can affect the antioxidant enzyme activity thus the thermotolerance of *L. edodes*. In this study, we used exogenous 2,4-D to increase the thermotolerance of *L. edodes* and explored the potentially molecular mechanism of 2,4-D enhanced thermotolerance in *L. edodes* by transcriptome analysis. 2,4-D improve *L. edodes’* thermotolerance possibly through regulating antioxidant genes, transcription factors, energy-provision system, membrane fluidity, cell wall remodeling, and other mechanisms. Future research can focus on functional analysis of the key genes through over-expression and gene editing. This study development of an potential molecular mechanism pathway to improve *L. edodes* thermotolerance through 2,4-D regulation and has also genetic basis for improving its thermotolerance, meanwhile, the results provided a reference for the research of auxin improving abiotic stress of fungi.

## Data Availability Statement

The original contributions presented in the study are publicly available. This data can be found at NCBI SRA (accession number: PRJNA81371).

## Author Contributions

YB, YZ, and YG conceived and designed the experiments. RX and SZ performed the experiments and wrote the paper. JS, HZ, and TZ contributed to modify and polish the paper. All authors contributed to the article and approved the submitted version.

## Funding

This research was funded by the National Natural Science Foundation of China (grant no. 31972476).

## Conflict of Interest

The authors declare that the research was conducted in the absence of any commercial or financial relationships that could be construed as a potential conflict of interest.

## Publisher’s Note

All claims expressed in this article are solely those of the authors and do not necessarily represent those of their affiliated organizations, or those of the publisher, the editors and the reviewers. Any product that may be evaluated in this article, or claim that may be made by its manufacturer, is not guaranteed or endorsed by the publisher.

## Supplementary Material

The Supplementary Material for this article can be found online at: https://www.frontiersin.org/articles/10.3389/fmicb.2022.910255/full#supplementary-material
