# Supplementary material for: Comparative Transcriptome Analysis Provides Insights Into the Mechanism by Which 2,4-Dichlorophenoxyacetic Acid Improves Thermotolerance in Lentinula edodes
Source: Front Microbiol. 2022 Jun 20;13:910255. doi: 10.3389/fmicb.2022.910255 (PMC9253865; doi:10.3389/fmicb.2022.910255)
Supplement: Supplementary file 1 [file Data_Sheet_1.docx]

Supplementary Material


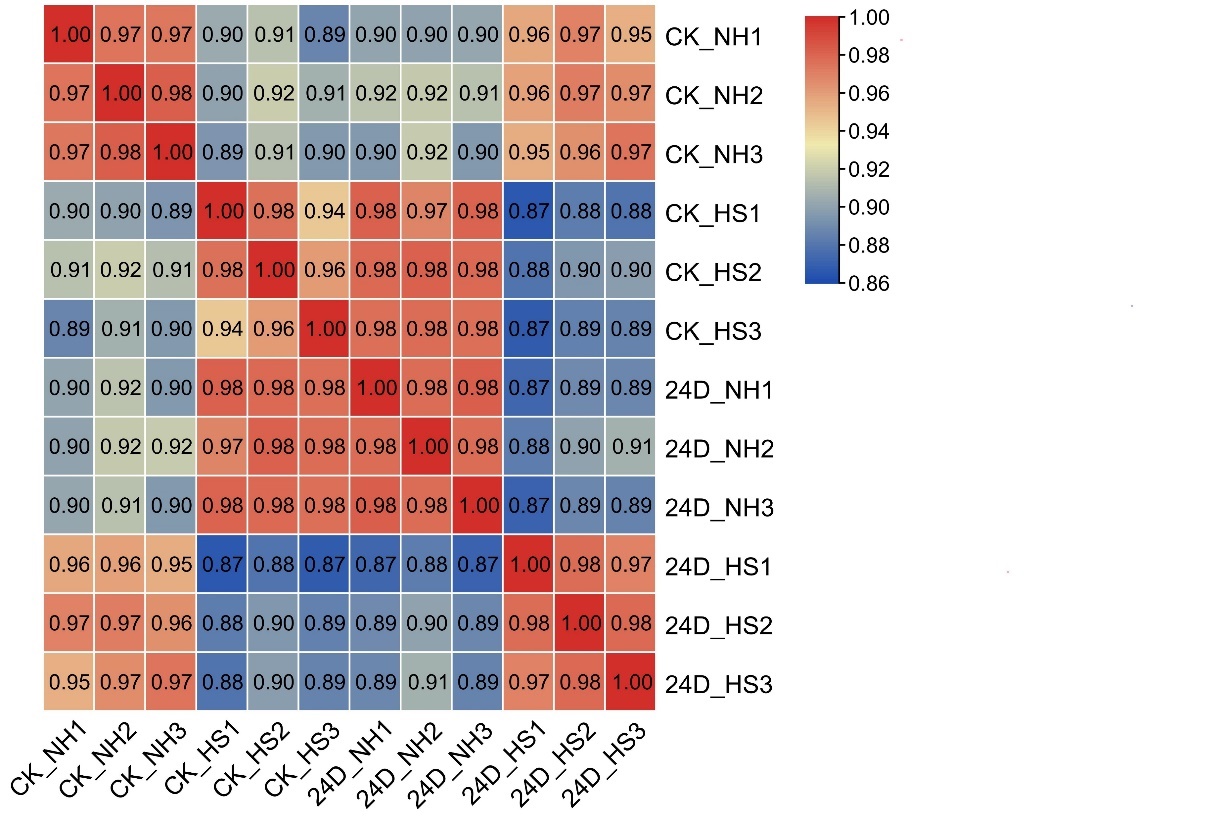


**Supplementary Figure S1.** Pearson's correlation coefficients for gene expression for different samples. CK_NH, CK_HS, 24D_NH and 24D_HS, respectively; 1, 2 and 3, three replicates per group. The values in the block chart represent the Pearson correlation coefficients for each of the two samples.


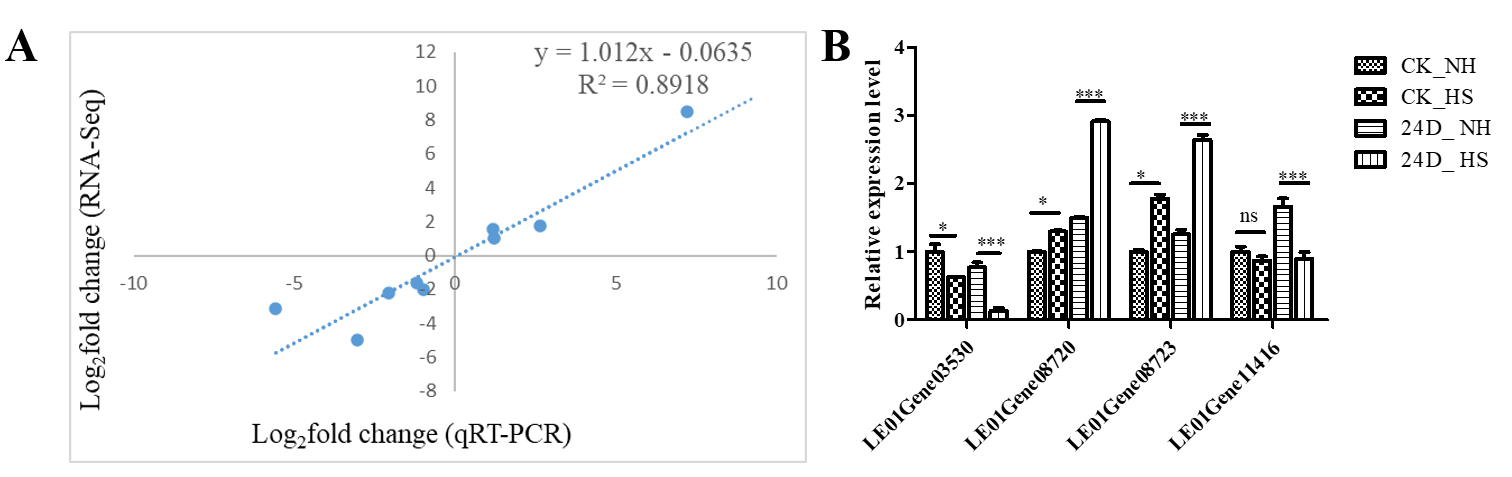


**Supplementary Figure S2.** qRT-PCR analysis **(A)** Relationship between RNA-seq and quantitative real-time PCR (qRT-PCR) expression data (log_2_fold change) (R^2^ = 0.8918). Nine genes were selected for qRT-PCR between 24D_HS/24D_NH samples, gene information is available in suptab1. The horizontal axis represents Log_2_fold change (RNA-Seq) and the vertical axis represents Log_2_fold change (qRT-PCR). 24D_NH and 24D_HS indicate exogenous 2,4-D, not heat stress and exogenous 2,4-D at heat stress, respectively. **(B)** showed the four genes qRT-PCR results (* *P <* 0.05; ** *P <* 0.01; *** *P <* 0.001; ns no significance).

**Supplementary Figure S3.** Effect of exogenous 2,4-D addition on the ATP content of *L. edodes* mycelium under heat stress. ATP content change of CK_NH, CK_HS, 24D_NH, and 24D_HS samples. These results are displayed as mean ± standard deviation (Bars, standard deviation of three replicates). Different letters mark significant differences between samples (*P* < 0.05).


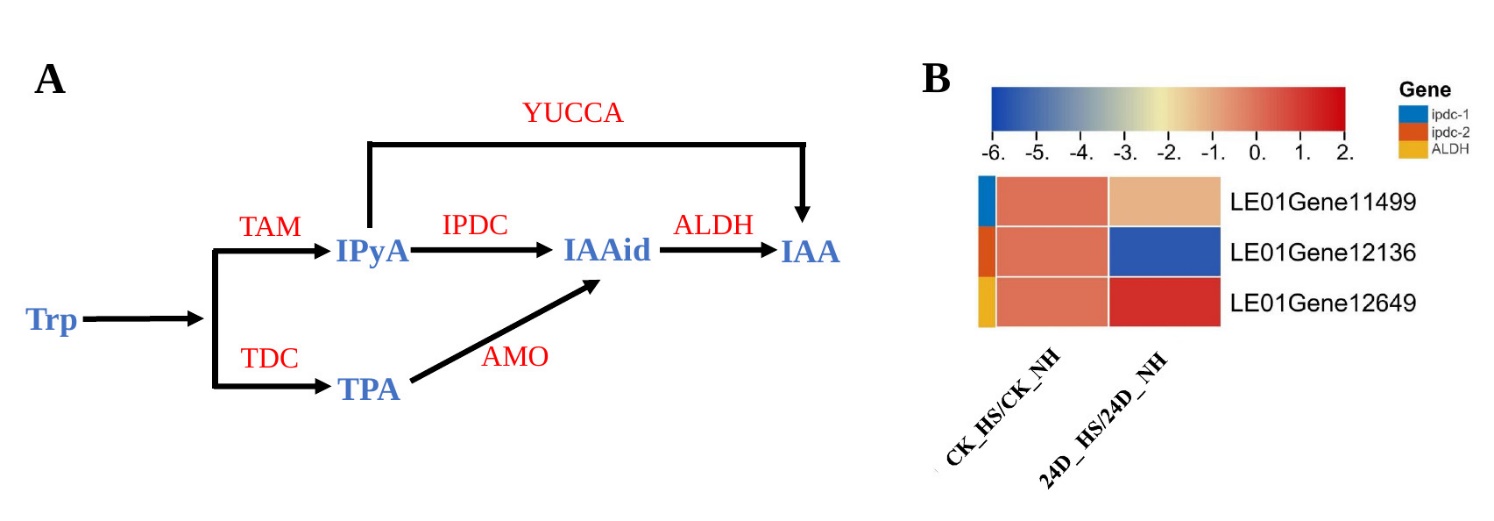


**Supplementary Figure S4.** The putative *L. edodes* IAA biosynthetic pathway and transcriptomic changes in related genes between CK_HS/CK_NH and 24D_ HS/24D_ NH. **(A)** YUCCA, IPA, and TPA pathways in *L. edodes*. TAM and YUCCA are the enzymes catalyzing the YUCCA pathway; TAM, IPDC, and are the enzymes catalyzing the IPyA pathway; TDC and AMO are the enzymes catalyzing the TPA pathway. Enzymes are in red font, metabolites are in blue font. Trp, Tryptophan; IPyA, Indole-3-pyruvate; TPA, Tryptamine; IAAid, Indole-3-acetaldehyde; IAA, indole-3-acetic acid. TAM, tryptophan aminotransferase; YUCCA, 3-indole pyruvate; IPDC, indole pyruvate decarboxylase; ALDH, aldehyde dehydrogenase; TDC, L-tryptophan decarboxylase; AMO, diamine oxidase. **(B)** Heat map showing the changes in expression of *ipc1*, *ipc2*, and *ALDH* genes between CK_HS/CK_NH and 24D_ HS/24D_ NH. Genes no differential expression are indicated by 0 in the heatmap.


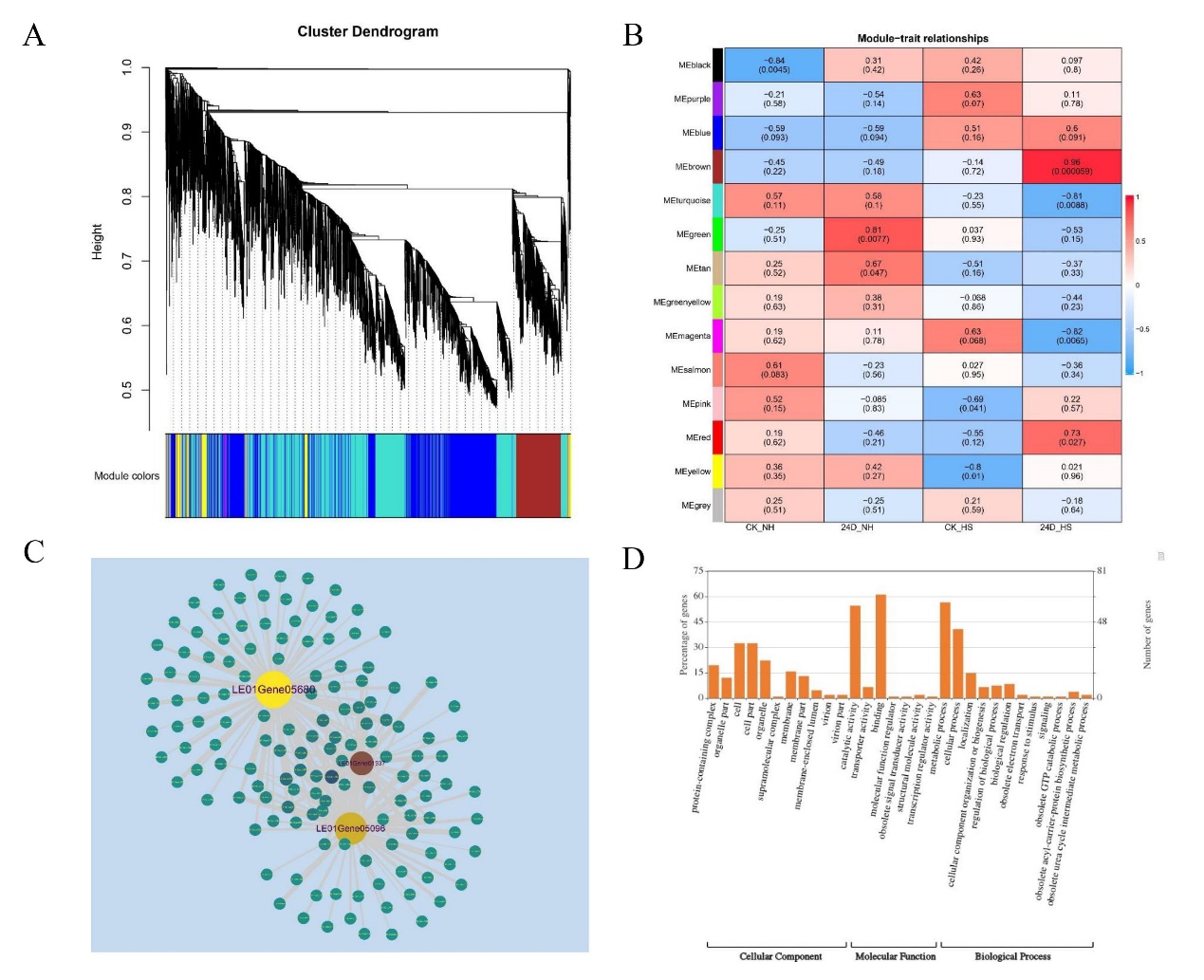


**Supplementary Figure S5.** Gene co-expression network analysis in CK_NH, CK_HS, 24D_NH, and 24D_HS samples. **(A)** The clustering dendrogram of WGCNA genes. The different color blocks at the bottom represent the different gene modules and the length of the block indicates the number of genes in each module. **(B)** Correlation diagrams of sample phenotypes and modules. The horizontal axis represents the phenotypes of the different groups. The vertical axis indicates the different gene modules. The numbers in the blocks represent the pearson correlation coefficients and *p* values (in brackets) included, respectively, where red represents the positive correlation between gene modules and blue indicates a negative correlation. **(C)** Gene co-expression network in the MEsaddlebrown module. Inside the circles are the gene names, whose sizes are indicated by the connectivity values, while the co-expression between two genes by straight lines. **(D)** Gene Ontology (GO) categories results of DEGs in MEsaddlebrown module, the horizontal axis represents the functional categories, and the vertical axis represents gene counts.

**Supplementary Table S1** Summary of the sequencing

| Sample name | Raw reads | Clean reads | Clean bases | Error rate(%) | Q20(%) | Q30(%) | GC(%) |
| --- | --- | --- | --- | --- | --- | --- | --- |
| CK_NH1 | 20,539,846 | 20,426,104 | 3.06G | 0.02 | 98.25% | 94.55% | 49.07% |
| CK_NH2 | 20,072,730 | 19,957,288 | 3.00G | 0.02 | 98.3% | 94.63% | 49.22% |
| CK_NH3 | 21,086,458 | 20,974,014 | 3.14G | 0.02 | 98.28% | 94.57% | 48.89% |
| CK_HS1 | 20,483,822 | 20,369,706 | 3.06G | 0.02 | 98.27% | 94.55% | 49.22% |
| CK_HS2 | 19,863,340 | 19,749,672 | 2.96G | 0.02 | 98.3% | 94.63% | 49.29% |
| CK_HS3 | 18,180,986 | 18,079,456 | 2.72G | 0.02 | 98.28% | 94.59% | 49.34% |
| 24D_NH1 | 20,433,420 | 20,314,128 | 3.04G | 0.02 | 98.26% | 94.53% | 48.84% |
| 24D_NH2 | 20,308,190 | 20,190,788 | 3.02G | 0.02 | 98.34% | 94.73% | 48.89% |
| 24D_NH3 | 20,269,130 | 20,156,882 | 3.02G | 0.02 | 98.37% | 94.83% | 48.70% |
| 24D_HS1 | 20,812,264 | 20,812,264 | 3.10G | 0.02 | 98.29% | 94.64% | 49.08% |
| 24D_HS2 | 20,812,264 | 19,633,006 | 2.94G | 0.02 | 98.40% | 94.86% | 48.96% |
| 24D_HS3 | 20,367,554 | 20,250,694 | 3.04G | 0.02 | 98.29% | 94.60% | 48.97% |

CK_NH1, CK_NH2, and CK_NH3: No heat stress treated samples (three independent biological replicates).

CK_HS1, CK_HS2, and CK_HS3: Heat stress treated samples (three independent biological replicates).

24D_NH1, 24D_NH2, and 24D_NH3: exogenous 2,4-D no heat stress treated samples (three independent biological replicates).

24D_HS1, 24D_HS2, and 24D_HS3: exogenous 2,4-D heat stress treated samples (three independent biological replicates).

Q20: The percentage of bases with a Phred value > 20.

Q30: The percentage of bases with a Phred value > 30.

**Supplementary Table S2** Unique GO term in 24D_ HS/24D_ NH up-regulated genes

| GO category | Gene ID | Gene annotation |
| --- | --- | --- |
| Supramolecular complex | LE01Gene01435 | α-tubulin |
|  | LE01Gene08622 | β-tubulin |
| Multicellular organismal process | LE01Gene07633 | Gonadotropin-releasing hormone |
| Obsolete GTP catabolic process | LE01Gene06942 | GTP-binding protein |
| Obsolete ATP-dependent proteolysis | LE01Gene10428 | Lon protease homolog |
|  | LE01Gene10711 | Lon protease homolog |
| Carbon utilization | LE01Gene10014 | Alanine aminotransferase |
|  | LE01Gene10979 | Phosphoenolpyruvate carboxykinase |
|  | LE01Gene11424 | NADP-dependent malic enzyme |

**Supplementary Table S3** Unique GO term in 24D_ HS/24D_ NH down-regulated genes

| GO category | Gene ID | Gene annotation |
| --- | --- | --- |
| Other organism part | LE01Gene08065 | DNA replication licensing factor |
| Obsolete gamma-glutamyltransferase Activity | LE01Gene02907 | Gamma-glutamyltranspeptidase |
| Obsolete electron transport | LE01Gene02033 |  |
|  | LE01Gene08705 | Peroxisomal acyl-coenzyme A oxidase |
|  | LE01Gene10455 | NADPH reductase |

**Supplementary Table S4** Primers used for qRT-PCR of selected differentially expressed genes

| Gene ID | Forward primer (5’-3’) | Reverse primer (5’-3’) | Gene annotation |
| --- | --- | --- | --- |
| LE01Gene00261 | GTCCAGAAGAGGGTGTTTATG | GCGGGATATGGCTTGTATAAC | Flavin-containing monooxygenase YUCCA |
| LE01Gene01273 | AGAGAAACCGCACGATACCT | ACCATGCCGTCTTTCCTGAT | LeDnaJ07 |
| LE01Gene02472 | GCGTGAGAAGGTCGATGATATT | TCGAGGAGGAGAAGGTCTTTAG | Hsp70 protein |
| LE01Gene03530 | ATGTCGTCTCTGAACCCTTATTC | TGACGTGGACGCATTGATAG | L-ascorbate peroxidase APX |
| LE01Gene06035 | AACGAGACAACCGAGCATAC | TACGTGCTCAGCTACCAAAC | No annotation |
| LE01Gene11499 | CTCGTCATCCTTCTCCCAATAAC | CGGGAAATGCTACCAACATAGA | indolepyruvate decarboxylase ipdC-1 |
| LE01Gene12136 | CCATCGCGGAGATTGTTAGAT | CCGCTCATACCCTTCACTAATC | indolepyruvate decarboxylase ipdC-2 |
| LE01Gene12260 | GTTGAACTCGGTGTTTCCTTTG | CTCCTGCGTTTGTTGGTAGA | Putative auxin transport protein |
| LE01Gene12649 | CTGGGCAATGGACGTTGATA | AATCCGGCCTCGTGAATAAG | Aldehyde dehydrogenase ALDH |
| Actin | GCATCCTGTCCTTCTTACCGAG | AAGAGCGAAACCCTCGTAGATG |  |

Actin represents the internal control gene.

**Supplementary Table S5** Biosynthesis of unsaturated fatty acids and fatty acid metabolism key genes change of CK_HS/CK_NH and 24D_ HS/24D_ NH

| Gene ID | Gene annotation | CK_HS/CK_NH | 24D_ HS/24D_ NH |
| --- | --- | --- | --- |
| LE01Gene01881 | 3-oxoacyl-[acyl-carrier-protein] reductase | n | 1.3886 |
| LE01Gene02324 | 3-ketoacyl-CoA thiolase | n | 1.407 |
| LE01Gene02815 | Delta(12) fatty acid desaturase | n | 1.1967 |
| LE01Gene02816 | Delta(12) fatty acid desaturase | n | 1.941 |
| LE01Gene02817 | Delta(12) fatty acid desaturase | n | 2.1129 |
| LE01Gene07809 | 3-hydroxy acyl-CoA dehydrogenase | n | 1.2359 |
| LE01Gene08296 | Acetyl-CoA acetyltransferase | n | 1.0068 |
| LE01Gene08705 | Peroxisomal acyl-coenzyme A oxidase | n | -1.3931 |
| LE01Gene12299 | Acylcarnitine carrier protein | n | 1.307 |

n represents genes no differential expression.

**Supplementary Table S6** Peroxisomal related genes change of CK_HS/CK_NH and 24D_ HS/24D_ NH

| Gene ID | Gene annotation | CK_HS/CK_NH | 24D_ HS/24D_ NH |
| --- | --- | --- | --- |
| LE01Gene01956 | Peroxisomal targeting signal receptor | n | 1.4861 |
| LE01Gene03405 | Peroxisomal membrane protein | n | 1.85 |
| LE01Gene07182 | Peroxisomal membrane protein | n | 1.2549 |
| LE01Gene07271 | Peroxisomal membrane protein | n | 1.0028 |
| LE01Gene04400 | Peroxisomal adenine nucleotide transporter | n | 2.2848 |

n represents genes no differential expression.

**Supplementary Table S7** Cell wall remodeling related genes change of CK_HS/CK_NH and 24D_ HS/24D_ NH

| Gene ID | Gene annotation | CK_HS/CK_NH | 24D_ HS/24D_ NH |
| --- | --- | --- | --- |
| LE01Gene10125 | Chitinase | n | 1.4513 |
| LE01Gene12054 | Chitosanase | n | 1.5285 |

n represents genes no differential expression.
